# Changes in Sepsis Biomarkers after Immunosuppressant Administration in Transplant Patients

**DOI:** 10.1155/2021/8831659

**Published:** 2021-01-05

**Authors:** Janka Franeková, Marek Protuš, Eva Kieslichová, Aleš Březina, Jitka Komrsková, Jiří Vymětalík, Antonín Jabor

**Affiliations:** ^1^Department of Laboratory Methods, Institute for Clinical and Experimental Medicine, Vídeňská 1958/9, 140 21 Prague 4, Czech Republic; ^2^Third Faculty of Medicine, Charles University, Ruská 87, 100 00 Prague 10, Czech Republic; ^3^Department of Anesthesiology, Resuscitation, and Intensive Care, Institute for Clinical and Experimental Medicine, Vídeňská 1958/9, 140 21 Prague 4, Czech Republic; ^4^First Faculty of Medicine, Charles University, Kateřinská 1660/32, 121 08 Prague 2, Czech Republic; ^5^Department of Anesthesiology and Resuscitation, Institute for Clinical and Experimental Medicine, Vídeňská 1958/9, 140 21 Prague 4, Czech Republic

## Abstract

Sepsis biomarkers change continuously during the postoperative period. We aimed to demonstrate the influence of immunosuppressants after transplantation (Tx) on presepsin, procalcitonin, CRP, white blood cells, and IL-6. A group of 140 patients after major surgery (86 non-Tx, 54 Tx) without any signs of sepsis or infectious complications was followed for 7 days. The changes in biomarkers were analyzed with respect to the type of surgery, organ, and induction immunosuppressant used (antithymocyte globulin, corticosteroids, or basiliximab/rituximab). Concentrations (95th percentiles) of presepsin and procalcitonin were higher in the Tx group (presepsin: Tx < 2380 vs. non‐Tx < 1368 ng/L, *p* < 0.05; procalcitonin: <28.0 vs. 3.49 *μ*g/L, *p* < 0.05). In contrast, CRP and IL-6 were lower in the Tx group (CRP: Tx < 84.2 vs. non‐Tx < 229 mg/L, *p* < 0.05; IL-6: <71.2 vs. 317 ng/L, *p* < 0.05). Decreases in CRP and IL-6 were found for all immunosuppressants, and procalcitonin was increased after antithymocyte globulin and corticosteroids. Negligible changes were found for white blood cells. Different responses of presepsin, procalcitonin, CRP, and IL-6 were therefore found in patients without any infectious complications after major surgery or transplantation. Immunosuppression decreased significantly IL-6 and CRP in comparison to non-Tx patients, while procalcitonin was increased after corticosteroids and antithymocyte globulin only. Cautious interpretation of sepsis biomarkers is needed in the early posttransplant period. This work was conducted as a noninterventional (nonregistered) study.

## 1. Introduction

Infection, documented or suspected, was the main diagnostic criterion of sepsis. White blood cells (leukocytes, WBC), plasma C-reactive protein (CRP), and procalcitonin (PCT) are listed as inflammatory variables in the International Guidelines for Management of Severe Sepsis and Septic Shock: 2012 (Surviving Sepsis Campaign) [1]. A new definition of sepsis has been suggested as “life-threatening organ dysfunction caused by a dysregulated host response to infection” (Sepsis-3), and acute changes to the SOFA score were recently recommended as a tool for the evaluation of organ dysfunction [2]. This recommendation considers the common SIRS criteria used to identify sepsis as unhelpful, and there is no single biomarker with the ability to distinguish between SIRS and sepsis [3].

Data describing cut-off values of infectious biomarkers in septic surgical patients are available. However, data describing discriminative values of presepsin (soluble CD14 subtype, PSEP), PCT, CRP, WBC, and interleukin 6 (IL-6) in surgical patients treated with immunosuppressants are lacking. Monitoring of postoperative values is essential for the detection of infection and sepsis. Concentrations of the respective biomarkers are time-dependent, even under uncomplicated conditions. Therefore, there is a need to define interpretative limits in situations without infectious complications.

We aimed to establish cut-off values of PSEP, PCT, CRP, WBC, and IL-6 in surgical patients during the first week after major surgery with respect to the type of induction immunosuppression treatment. We present the data of the first part of the study describing time-dependent reference limits of PSEP, PCT, CRP, WBC, and IL-6 in surgical patients without any infectious complications during the first postoperative week.

## 2. Material and Methods

The measurement and evaluation of the decision limits, the dynamic changes of inflammatory biomarkers, and the influence of immunosuppressants were undertaken in a single-center, noninterventional, prospective, observational study in patients undergoing major surgery admitted to the Department of Anesthesiology and Resuscitation, Institute for Clinical and Experimental Medicine (IKEM), Prague, Czech Republic.

### 2.1. Patients

A group of 140 patients was evaluated. Inclusion criteria were consecutive adult patients who underwent major cardiac or abdominal surgery, including transplantation of solid organs (kidney, liver, and pancreas), survival up to 30 days after surgery, and written informed consent.

Exclusion criteria were any clinical sign of the systemic or local infection in the early postoperative period up to 7 days after surgery, age below 18 years, and disagreement with participation in the study.

Two subsets of patients were evaluated. The first subset (control group, non-Tx) consisted of 86 patients after major abdominal (large or small intestine surgery in 25 patients, resection of the liver or stomach in 20 patients, pancreatectomy in 12 patients, aorta reconstruction in 11 patients, prostate surgery in 3 patients, and kidney resection in 1 patient) and cardiac surgeries (aortocoronary bypass or valve reconstruction in 14 patients). The second subset (Tx) consisted of 54 patients who underwent solid organ transplantation (kidney Tx in 31 patients, liver Tx in 16 patients, and pancreas or kidney plus pancreas Tx in 7 patients). Induction immunosuppression (antithymocyte globulin, basiliximab, rituximab, infliximab, or corticosteroids) was administered in addition to a standard regimen with corticosteroids, calcineurin inhibitors, and mycophenolate mofetil.

All surgeries were performed at IKEM, Czech Republic.

### 2.2. Sampling

Blood samples were taken immediately before the surgery, at the 3rd hour after surgery and on the 1st, 2nd, 3rd, 5th, and 7th days following the surgery. The plasma samples were stored at -70°C until analysis.

### 2.3. Laboratory Methods

All measurements were performed in the accredited laboratory (ISO 15189) of the IKEM Department of Laboratory Methods. EDTA plasma (for the measurement of PSEP), lithium heparin plasma (for the measurement of PCT, CRP, and IL-6), and EDTA tubes for whole blood (for the measurement of WBC) were used according to the manufacturer's instructions (Greiner Vacuette system, Greiner Bio-One North America, Monroe, North Carolina, USA).

PSEP (reference range of 60 to 365 ng/L) was measured on a Mitsubishi Pathfast analyzer using a Pathfast Presepsin kit (Mitsubishi Chemical Medience Corporation, Tokyo, Japan).

PCT (reference range, as specified by the manufacturer, was <0.5 *μ*g/L) and IL-6 (reference range, as specified by the manufacturer, was <7 ng/L) were measured on a Roche Cobas 6000 analyzer (Roche Diagnostics, Rotkreuz, Switzerland) with the Elecsys Brahms Procalcitonin kit (Roche Diagnostics, Mannheim, Germany) and Elecsys IL-6 kit (Roche Diagnostics, Mannheim, Germany).

CRP (reference range ≤ 5 mg/L) was measured on an Abbott Architect analyzer (Abbott Laboratories, Abbott Park, Illinois, USA) with a Sentinel CRP Vario kit (Sentinel Diagnostics, Milan, Italy).

WBC in whole blood (EDTA, Greiner Vacuette system) was measured on a Sysmex XE5000 analyzer (Sysmex Europe GmbH, Norderstedt, Germany) immediately after sampling.

Quality control was performed using the Pathfast Presepsin Control, Roche PreciControl PCT, Roche PreciControl Multimarker, and Bio-Rad Liquichek Immunology (Bio-Rad Laboratories, Hercules, California, USA) commercial control materials.

### 2.4. Statistical Methods

Medians and nonparametric determinations of percentiles (5th, 10th, 25th, 75th, 90th, and 95th percentiles, MedCalc software, version 18.11.6) were used, the Mann-Whitney test for independent populations was used for the comparison of the evaluated groups of patients, and the Bonferroni correction was used for multiple comparisons. The chi-square test was used for the comparison of proportions.

## 3. Results

Detailed characteristics of all patients and patients in the non-Tx and Tx subsets are given in Table 1.

All patients were without any detected systemic or local infection up to 7 days after surgery. The number (%) of patients without infection at other time points (10th day, day of discharge, and 30th day) is displayed. Statistical difference between non-Tx and Tx patients is also given. The Tx patients were younger with a worse APACHE score. However, the length of surgery was shorter (Table 1). All patients survived 30 days, and there were no cases of sepsis, septic shock, peritonitis, or other infectious complications during the first postoperative week, including local infection.

Detailed information on the induction immunosuppressant regimens that were used is given in Table 2. All transplant patients were treated with standard long-term antirejection therapy, which includes a combination of corticosteroids, calcineurin inhibitors, and mycophenolate mofetil.

**Figure 1:**
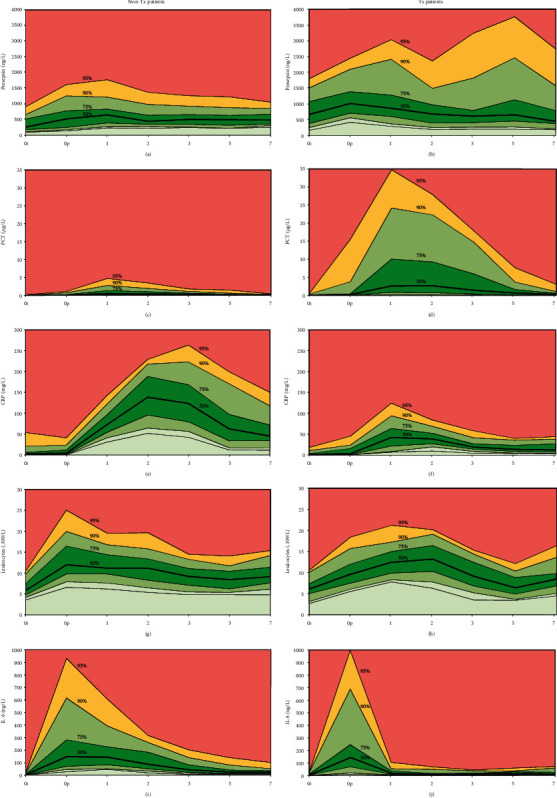
Concentrations of PSEP, PCT, CRP, leukocytes, and IL-6 in a group of patients without immunosuppressants after major surgery ((a, c, e, g, i) non-Tx patients) and in patients with immunosuppressants after Tx of solid organs ((b, d, f, h, j) Tx patients).

Figure 1 describes the results of the measurements at defined time points before and after surgery for the non-Tx and Tx subsets of patients. Only patients without any signs of septic complications or infection (up to 7 days after surgery) were evaluated. Therefore, these data are considered a model of postoperative noninfectious systemic inflammatory response.

The interquartile range is filled with green, values up to the 25th and between the 75th and 90th percentiles are filled with light green, values above the 90th percentile are filled with yellow, and values above the 95th percentile are filled with light red. The bold line represents median values. Similar ranges are used in the *y*-axes for the non-Tx and Tx patients to highlight the differences between both groups.

Similarly, Supplementary Table 1 describes measured concentrations (median, 5th and 95th percentiles) and statistically significant differences between non-Tx and Tx subsets for data presented in Figure 1. Supplementary Table 2 describes the results for the entire group of 140 patients (median, 5th and 95th percentiles).

All tested biomarkers (PSEP, PCT, CRP, WBC, and IL-6) were significantly influenced by postoperative stress in patients without any infectious complications. PSEP and PCT displayed peak concentrations during the 1st day in both the non-Tx and Tx patients, and both biomarkers were significantly lower in the non-Tx patients. The concentrations of PCT were extremely high in some of the Tx patients.

CRP and IL-6 displayed a similar response in Tx patients with significantly lower concentrations in comparison to the non-Tx subset of patients. However, the time response was different: peak values of IL-6 were observed immediately after surgery, and CRP responded with a delay. Peak concentrations of CRP were found on the 2nd to 3rd day in the non-Tx patients but on the 1st day in the Tx patients.

Leukocyte concentrations displayed rather flat changes over time with an early peak in the non-Tx patients (immediately after surgery) and a delayed peak in the Tx patients (peak between the 1st and 2nd days after surgery).

**Figure 2:**
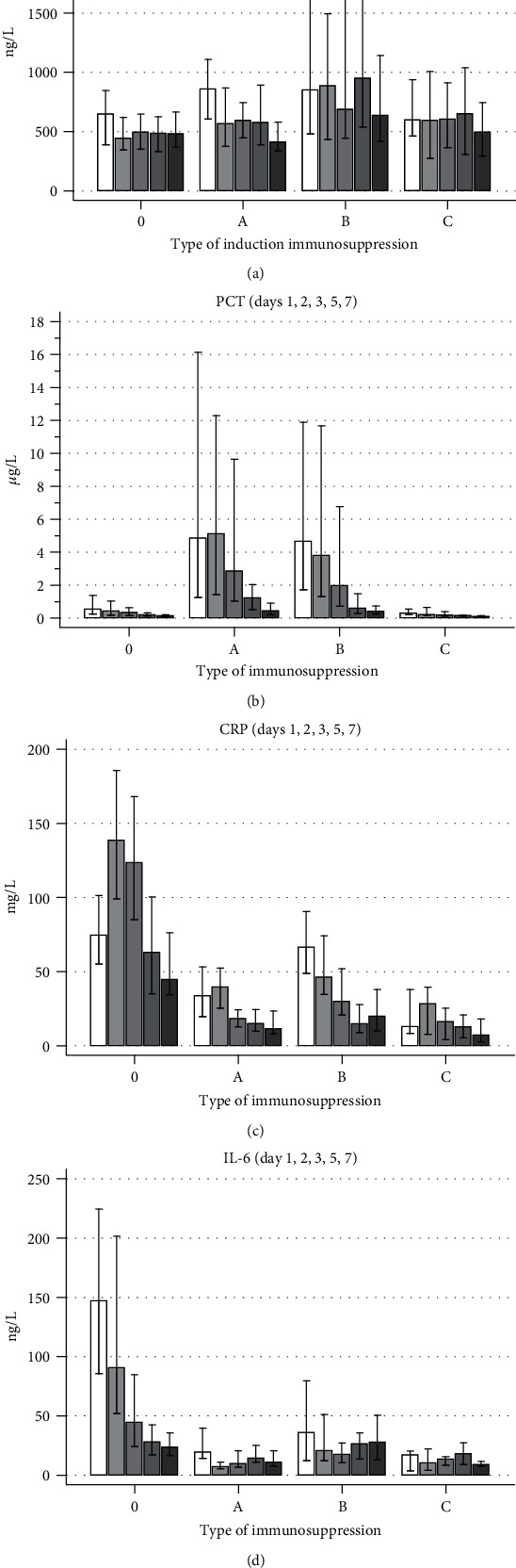
Analysis of the influence of different immunosuppressant regimens on (a) PSEP, (b) PCT, (c) CRP, and (d) IL-6. Medians and interquartile ranges are given. Immunosuppressants used: 0 = none, A = standard regimen plus ATG, B = standard regimen plus corticosteroid induction, and C = standard regimen with basiliximab and rituximab (for details, see Table 2). The five columns represent postoperative days 1, 2, 3, 5, and 7 (from light to dark).

The influence of different immunosuppressants in comparison to the non-Tx control subset of patients is given in Figure 2. Three groups of induction immunosuppressant therapy in addition to the standard regimen (corticosteroids, tacrolimus, and mycophenolate mofetil) were compared: (a) antithymocyte globulin (ATG) (with or without infliximab), (b) corticosteroid induction, and (c) basiliximab and/or rituximab without ATG. The highest concentrations of PCT after Tx were found after administration of ATG and in patients with corticosteroid induction. Conversely, CRP and IL-6 were lower in all immunosuppressant regimens after comparison of Tx and non-Tx patients. Supplementary Table 3 describes significant differences of immunosuppressant regimens during the 1st to 7th posttransplant day.

The separate analysis of biomarkers in patients with kidney, liver, and kidney plus pancreas transplantation is given in Supplementary Tables 4 and 5. In comparison to non-Tx patients, a similar response in all types of organ transplantation was found for CRP and IL-6 (both decreased during the postoperative course in comparison to the non-Tx subset), PCT (increased during the postoperative course), and WBC (occasional or quantitatively negligible changes). However, PSEP was higher during the postoperative course only in patients with liver Tx in comparison to the non-Tx group (Supplementary Table 5).

## 4. Discussion

We described time-dependent limits for the interpretation of five biomarkers (PSEP, PCT, CRP, WBC, and IL-6), which can be used as acceptable values in uncomplicated patients without infection after major surgery, including patients after kidney, liver, and pancreas transplantation. We found important changes during the postoperative course and differences between subsets of patients with and without immunosuppressant administration.

WBC has been traditionally used as a biomarker for SIRS with specific limits (WBC count > 12 × 10^9^/L or <4.0 × 10^9^/L or more than 10% of immature forms in the case of normal WBC count) since 1992 [4]. In our study, an increase in leukocyte levels was found early after surgery, within the 1st day with values lower than 20 × 10^9^/L in more than 95% of patients. The use of WBC is less specific and less sensitive for the detection of septic complications. However, the influence of immunosuppressant administration on WBC was less pronounced in comparison to the influence of immunosuppression on PCT, CRP, and IL-6.

PCT has been used as a sepsis biomarker with medium size sensitivity and specificity [5]. However, PCT should be used together with clinical and other laboratory data [1, 6]. Moreover, PCT is influenced by ATG administration during the early posttransplant period [7]. This limitation makes PCT almost useless in transplant surgery. Similar changes were found in our study: more than half of the Tx patients exhibited PCT concentrations above 2.8 *μ*g/L on the 1st day after transplantation. In contrast, in the non-Tx patients without administration of ATG, only 10% of patients exhibited PCT concentrations above this value (2.8 *μ*g/L) on the 1st day after surgery. In summary, any concentration of PCT above 2.8 *μ*g/L can be regarded as an undoubted pathology only in patients without induction with ATG or corticosteroid administration.

CRP has been used for years as a biomarker of septic complications after major surgery with a simple interpretative equation that describes an upper acceptable limit (in mg/L) of 500/*d*, where *d* is the number of days after surgery and *d* ≥ 5 [8]. However, such limits depend on the studied population and the type and extent of surgery [9]. Adamina et al. found, in their meta-analysis of 1986 patients, lower cut-off limits in patients with laparoscopic abdominal surgery (the 4th postoperative day: cut-offs 56–74 mg/L with sensitivity to detect infection 77–100%, specificity 49–66%, positive predictive value (PPV) of 45% to 48%, and negative predictive value (NPV) of 88% to 100%) than in those with open abdominal surgery (cut-offs 110–184 mg/L with sensitivity of 50% to 100%, specificity 67–86%, PPV 46–69%, and NPV 79–100%) [10]. A high increase in CRP with a delayed peak was found in our patients with a significant difference between the non-Tx and Tx patients (Figure 1, Supplementary Table 1): peak concentrations of CRP were approximately two-fold lower in the Tx patients than in the non-Tx patients. The reason for the lower CRP concentrations could be two-fold: decreased values of IL-6 after immunosuppressant administration (Figure 2) or decreased liver function in patients who underwent liver transplantation. However, it was difficult to estimate suitable and time-specific cut-off limits of CRP during the postoperative period in the non-Tx and Tx patients. Therefore, the ability of CRP to identify patients with infectious complications is compromised, monitoring is essential, and the use of other biomarkers is advisable.

Concentrations of PSEP, a novel sepsis biomarker, were significantly increased in patients with sepsis, severe sepsis, or septic shock [11–15] and in patients with infectious complications after cardiac surgery [16], including heart transplantation [17]. Zhang et al. [18], Wu et al. [19], and Romualdo et al. [20] reported data on the sensitivity and specificity of PSEP cut-offs in patients with infection and SIRS. However, PSEP concentrations are also influenced by SIRS, renal damage, and ages above 70 years [21], and no superiority above other biomarkers was found in intensive care patients [22]. In our study, PSEP was significantly higher before surgery in patients with solid organ transplantation. However, the relative increase was lower in these patients in comparison to preoperative values (Figure 1, Supplementary Table 1). An absolutely “safe” limit for PSEP appears to be approximately 1000 ng/L in patients without immunosuppressant administration and approximately 1500 ng/L in Tx patients. The kidney function should be taken into consideration. However, we were unable to determine a specific response in patients with kidney Tx.

There was a multiple effect of immunosuppressant regimens on biomarker concentrations (Figure 2). We found a significant increase in PCT after ATG administration. This is a known phenomenon and one of the clinical disadvantages of PCT measurement [7]. However, increased PCT was also found in patients who received corticosteroid induction in addition to the standard regimen (corticosteroids, tacrolimus, and mycophenolate mofetil). On the contrary, PCT concentrations in patients with basiliximab and rituximab were comparable to those in the non-Tx subset. A uniform response of CRP and IL-6 was found in all Tx patients with any type of induction immunosuppression: significantly decreased concentrations in the early posttransplant course in comparison to non-Tx patients. There was no difference between PSEP and WBC in Tx patients in comparison to non-Tx patients without immunosuppression. The use of CRP and IL-6 as biomarkers of sepsis or infection after solid organ Tx is therefore doubtful, and PCT should be used with caution only in specific groups of patients (e.g., induction immunosuppression with basiliximab and rituximab).

We also analyzed the relationship between the type of operation and changes in respective biomarkers (Supplementary Tables 4 and 5). The uniform decrease in CRP and IL-6 was found in all types of organ Tx, even in patients with longer surgery time or higher APACHE II score. Surprisingly, in spite of increased creatinine after the kidney or kidney plus pancreas Tx, the concentration of PSEP was increased more frequently in patients after liver Tx. However, concentrations of PCT and PSEP were positively correlated with serum creatinine in both non-Tx and Tx patients.

### 4.1. Limitations of the Study

The first part of our study was focused on carefully selected patients without any infectious complications after major surgery. Despite the exclusion criteria, the main limitation of this study was the heterogeneity of the evaluated population and the limited number of patients. A certain limitation was that patients without systemic or local infectious complications up to 7 days after surgery were included. Therefore, infection could appear subsequently in patients. However, up to 10 days after surgery, local infection was found in only 3 patients (Table 1). Certain limitation was the difference of the APACHE II score between Tx and non-Tx patients. However, it is impossible to recruit comparable patients at least due to the age (higher in non-Tx patients) and organ failure (always present in Tx patients). Conversely, an advantage of our study protocol was the frequency of sampling, which was sufficiently high to assess the changes in biomarker concentrations. Here, we present data of the noninfected “reference” surgical population. The second part of our study will be focused on septic patients with the aim of establishing cut-off values to rule in or rule out infection in surgical patients treated with immunosuppressants.

## 5. Conclusions

All of the tested biomarkers (PSEP, PCT, CRP, WBC, and IL-6) were significantly influenced by postoperative stress in patients without any infectious complications. The increase in PSEP was lower in comparison to PCT, CRP, and IL-6, and peak values of PSEP were found on the 1st day. The Tx patients exhibited higher concentrations of PSEP than non-Tx patients. Peak values of PCT were found on the 1st day after operation with significantly increased values in patients with immunosuppressant administration. However, there were individual differences in the relative increase in PCT compared to the preoperative values. CRP responded with delay (peak on the 2nd to 3rd day), and considerably lower concentrations were found in the transplanted patients. A very flat response was found for WBC, and no specific difference was found between the non-Tx and Tx patients. Dynamic changes of IL-6 became evident very early after surgery (peak immediately after operation). However, the concentrations of IL-6 were significantly lower in patients with immunosuppressant administration. No specific cut-off limit could be defined for all tested biomarkers.

In summary, time-dependent and immunosuppressant-specific decision limits are needed for the proper interpretation of inflammatory biomarkers after major surgery. Uncoupled response of inflammatory biomarkers after Tx is caused probably by the immunosuppression. IL-6 and CRP were significantly lower after induction immunosuppression with ATG or corticosteroids, or basiliximab/rituximab, when compared to the control group after major surgery without immunosuppressants. On the contrary, PCT increases without any signs of infection or sepsis after ATG and corticosteroids, but not after basiliximab/rituximab induction immunosuppression. Minimal influence of immunosuppressant was found for WBC and PSEP. Frequent monitoring regimens and the use of multiple biomarkers are advisable.

## Figures and Tables

**Table 1:** Patient characteristics.

	All (*N* = 140)	Non-Tx (*N* = 86)	Tx (*N* = 54)	Statistical difference between non-Tx and Tx
Age (years)	64.0 (50.5–70.5)	65.5 (59.0–73.0)	54.5 (43.0–65.0)^†^	*p* < 0.001
Men (*N*, %)	90 (64%)	51 (59%)	39 (72%)	N.S.
APACHE II	11 (7.0–14.0)	9 (6.0–13.0)	13 (10.0–15.0)^†^	*p* < 0.001
Length of surgery (min)	225 (180–284)	230 (185–295)	190 (160–270)^†^	*p* < 0.05
Blood loss (mL)	200 (100–575)	200 (100–600)	200 (100–500)	N.S.
Length of stay in ICU (days)	11.0 (9.0–14.0)	9.0 (7.0–12.0)	13.0 (11.0–15.0)^†^	*p* < 0.001
Without any infection up to 10 days after surgery (*N*, %)	137 (98%)	84 (98%)	53 (98%)	N.S.
Without any infection till discharge (*N*, %)	127 (91%)	81 (94%)	46 (85%)	N.S.
Without any infection up to 30 days after surgery (*N*, %)	119 (85%)	76 (88%)	43 (80%)	N.S.

Age, APACHE II, length of surgery/stay in ICU, and blood loss are given as median (interquartile range). ^†^Significantly different values between the non-Tx and Tx patient subsets (*p* < 0.05).

**Table 2:** Immunosuppressant therapy in the group of 54 Tx patients.

Transplanted organ	Number of patients	Induction immunosuppression (in addition to a standard regimen with corticosteroids, calcineurin inhibitors, and mycophenolate mofetil)
Kidney	21	Antithymocyte globulin
	5	Basiliximab
	3	Antithymocyte globulin+infliximab
	2	Basiliximab+rituximab

Liver	15	Corticosteroids
	1	Basiliximab

Pancreas	1	Antithymocyte globulin
	1	Basiliximab

Kidney plus pancreas	5	Antithymocyte globulin

## Data Availability

All data are stored with the corresponding author.

## Supplementary Materials

Supplementary 1 Supplementary Table 1: a model of noninfectious inflammatory response—the comparison between non-Tx and Tx subsets of patients.

Supplementary 2 Supplementary Table 2: a model of noninfectious inflammatory response—all patients.

Supplementary 3 Supplementary Table 3: the relationship between biomarker changes and three immunosuppressant regimens used (ATG, corticosteroid, and basiliximab/rituximab).

Supplementary 4 Supplementary Table 4: patient characteristics with respect to the type of surgery.

Supplementary 5 Supplementary Table 5: the relationship between biomarker changes and the type of surgery.
