# Supplementary material for: Changes in Sepsis Biomarkers after Immunosuppressant Administration in Transplant Patients
Source: Mediators Inflamm. 2021 Jan 5;2021:8831659. doi: 10.1155/2021/8831659 (PMC7811562; doi:10.1155/2021/8831659)
Supplement: Supplementary 1 — Supplementary Table 1: a model of noninfectious inflammatory response—the comparison between non-Tx and Tx subsets of patients. [file 8831659.f1.docx]

**Supplementary Table 1**

The comparison between Non-Tx (N=86) and Tx (N=54) subsets of patients. Model of non-infectious inflammatory response. Plasma concentrations of presepsin, PCT, CRP, leukocytes, and IL-6 were measured before and after transplantation of liver and kidney. Data are presented as the median (5th – 95th interval). The medians in bold with asterisk show significant differences (after the Bonferroni correction) in comparison to the non-Tx group of patients. See also Figure 1.

| Time points and statistics | | **Presepsin (ng/L)** | | **PCT (µg/L)** | | **CRP (mg/L)** | | **Leukocytes (x 10^9^/L)** | | **IL–6 (ng/L)** | |
| --- | --- | --- | --- | --- | --- | --- | --- | --- | --- | --- | --- |
|  |  | **Non–Tx** | **Tx** | **Non–Tx** | **Tx** | **Non–Tx** | **Tx** | **Non–Tx** | **Tx** | **Non–Tx** | **Tx** |
| 0 | Median | 264 | **672*** | 0.05 | **0.14*** | 3.0 | 1.8 | 5.8 | 6.1 | 4.5 | 5.9 |
|  | 90% CI | (97.0 – 908.0) | (164.8 – 1801.0) | (0.02 – 0.23) | (0.03 – 0.50) | (0.38 – 54.2) | (0.31 – 18.4) | (3.57 – 10.5) | (2.63 – 10.5) | (2.21 – 21.8) | (2.40 – 28.0) |
| +3 h | Median | 523.0 | **1024.0*** | 0.12 | **0.31*** | 4.15 | 3.3 | 12.0 | **9.7*** | 147.7 | 146.6 |
|  | 90% CI | (141.4 – 1617.4) | (414.3 – 2454.4) | (0.02 – 1.16) | (0.10 – 16.2) | (0.68 – 41.4) | (0.60 – 45.8) | (6.58 – 25.2) | (5.52 – 18.5) | (27.8 – 931.6) | (10.5 – 1016.2) |
| +1 d | Median | 643.0 | **874.5*** | 0.56 | **3.05*** | 74.1 | **42*** | 11.2 | 12.3 | 144.5 | **18.9*** |
|  | 90% CI | (234.4 – 1779.8) | (286.4 – 3038.0) | (0.07 – 4.75) | (0.19 – 35.2) | (31.1 – 143.4) | (7.06 – 124.7) | (6.18 – 19.6) | (7.78 – 21.3) | (46.6 – 606.3) | (4.40 – 106.8) |
| +2 d | Median | 447.0 | 682.5 | 0.45 | **2.76*** | 138.6 | **38.7*** | 11.2 | **12.3*** | 87.4 | **9.3*** |
|  | 90% CI | (218.6 – 1368.0) | (188.4 – 2379.8) | (0.07 – 3.49) | (0.17 – 28.0) | (51.9 – 229.3) | (9.56 – 84.2) | (5.40 – 19.7) | (6.25 – 20.2) | (18.8 – 316.8) | (3.74 – 71.2) |
| +3 d | Median | 502.0 | 618.0 | 0.37 | **1.68*** | 122.9 | **19.3*** | 9.2 | 9.0 | 43.5 | **12.2*** |
|  | 90% CI | (245.0 – 1262.3) | (196.0 – 3251.6) | (0.07 – 1.79) | (0.12 – 17.9) | (41.8 – 264.8) | (4.40 – 58.9) | (4.84 – 14.3) | (3.49 – 15.3) | (7.41 – 202.3) | (4.50 – 45.5 |
| +5 d | Median | 500.0 | **651.0*** | 0.22 | **0.76*** | 62.8 | **14.1*** | 8.45 | **6.2*** | 26.6 | **19.7*** |
|  | 90% CI | (218.2 – 1225.1) | (194.4 – 3782.8) | (0.04 – 1.53) | (0.08 – 7.82) | (12.2 – 199.6) | (3.80 – 40.3) | (4.85 – 14.1) | (3.41 – 12.2) | (8.83 – 140.1) | (5.03 – 60.8) |
| +7 d | Median | 483.5 | 443.5 | 0.15 | **0.35*** | 45.1 | **11.9*** | 9.1 | 8.5 | 23.6 | **12.6*** |
|  | 90% CI | (255.0 – 1056.5) | (180.9 – 2743.7) | (0.05 – 0.45) | (0.06 – 2.91) | (11.5 – 151.3) | (2.52 – 44.4) | (4.73 – 15.4) | (4.43 – 16.4) | (9.51 – 101.8) | (5.04 – 72.4) |
