# Supplementary material for: Changes in Sepsis Biomarkers after Immunosuppressant Administration in Transplant Patients
Source: Mediators Inflamm. 2021 Jan 5;2021:8831659. doi: 10.1155/2021/8831659 (PMC7811562; doi:10.1155/2021/8831659)
Supplement: Supplementary 2 — Supplementary Table 2: a model of noninfectious inflammatory response—all patients. [file 8831659.f2.docx]

**Supplementary Table 2**

All patients, N=140, model of non-infectious inflammatory response. Plasma concentrations of presepsin, PCT, CRP, leukocytes, and IL-6 were measured before and after major surgery. Data are presented as the median (5th – 95th interval).

| Time points | Presepsin  (ng/L) | PCT  (µg/L) | CRP  (mg/L) | Leukocytes  (x 10^9^/L) | IL-6 (ng/L) |
| --- | --- | --- | --- | --- | --- |
| Before surgery | 377.5  (105.9 – 1593.6) | 0.08  (0.02 – 0.38) | 2.4  (0.33 – 41.1) | 5.90  (3.20 – 10.5) | 5.35  (2.31 – 27.6) |
| +3 hours after surgery | 674.0  (165.4 – 2090.8) | 0.17  (0.03 – 3.31) | 3.8  (0.60 – 42.2) | 11.1  (5.99 – 22.8) | 147.6  (14.7 – 939.4) |
| +1 day after surgery | 709.0  (259.0 – 2502.6) | 1.04  (0.09 – 21.6) | 63.4  (10.7 – 138.4) | 11.5  (6.98 – 20.0) | 56.9  (7.58 – 476.6) |
| +2 days after surgery | 531.5  (196.7 – 1977.4) | 0.84  (0.09 – 21.2) | 80.5  (15.8 – 224.4) | 11.8  (5.73 – 19.9) | 43.1  (4.23 – 262.1) |
| +3 days after surgery | 532.5  (231.8 – 1983.8) | 0.47  (0.08 – 11.3) | 73.5  (8.15 – 233.6) | 9.2  (4.62 – 14.9) | 24.1  (5.00 – 148.3) |
| +5 days after surgery | 563.5  (211.2 – 2599.2) | 0.31  (0.05 – 3.75) | 30.7  (4.51 – 177.0) | 7.5  (3.72 – 12.4) | 22.2  (6.82 – 95.5) |
| +7 days after surgery | 480.0  (211.3 – 1465.5) | 0.19  (0.05 – 1.01) | 29.5  (4.27 – 119.5) | 8.85  (4.40 – 15.6) | 18.8  (6.16 – 76.0) |
