# Supplementary material for: Changes in Sepsis Biomarkers after Immunosuppressant Administration in Transplant Patients
Source: Mediators Inflamm. 2021 Jan 5;2021:8831659. doi: 10.1155/2021/8831659 (PMC7811562; doi:10.1155/2021/8831659)
Supplement: Supplementary 4 — Supplementary Table 4: patient characteristics with respect to the type of surgery. [file 8831659.f4.docx]

**Supplementary Table 4**

Patient characteristics with respect to the type of surgery. Age, length of surgery and ICU stay, and APACHE II are presented as the medians. Statistical difference is given in comparison to the non-Tx group (p<0.05). For details see also Table 1.

|  | Non-Tx  (N=86) | Kidney Tx (N=31) | Liver Tx (N=16) | Kidney plus pancreas Tx (N=7) |
| --- | --- | --- | --- | --- |
| Age (years) | 64.0 | 52.0 (p<0.05) | 65.5 (N.S.) | 44.0 (p<0.05) |
| Length of the surgery (min) | 225 | 170 (p<0.05) | 249 (N.S.) | 350 (p<0.05) |
| Blood loss (mL) | 200 | 100 (p<0.05) | 1300 (p<0.05) | 400 (N.S.) |
| Length of ICU stay (days) | 11 | 13.0 (p<0.05) | 13.5 (p<0.05) | 13 (p<0.05) |
| APACHE II | 11 | 12.5 (p<0.05) | 13.5 (p<0.05) | 13 (p<0.05) |
