# Supplementary material for: Changes in Sepsis Biomarkers after Immunosuppressant Administration in Transplant Patients
Source: Mediators Inflamm. 2021 Jan 5;2021:8831659. doi: 10.1155/2021/8831659 (PMC7811562; doi:10.1155/2021/8831659)
Supplement: Supplementary 5 — Supplementary Table 5: the relationship between biomarker changes and the type of surgery. [file 8831659.f5.docx]

**Supplementary Table 5**

The relationship between biomarker changes and the type of surgery. The non-Tx group (N=86) was taken as a reference. The biomarkers were tested each day during the early posttransplant period in comparison to the non-Tx group. In case of significant increase or decrease (p<0.01) in the concentration of the respective biomarker, number of the particular day is given (Day No.). The Bonferroni correction was applied.

|  | Kidney Tx (N=31) | Liver Tx (N=16) | Kidney plus pancreas Tx (N=5) |
| --- | --- | --- | --- |
| Increased presepsin (Day No.) | 1 | 1,2,3,5,7 | - |
| Decreased presepsin (Day No.) | - | - | - |
| Increased PCT (Day No.) | 1,2,3,5,7 | 1,2,3,5,7 | 1,2,3,5,7 |
| Decreased PCT (Day No.) | - | - | - |
| Increased CRP (Day No.) | - | - | - |
| Decreased CRP (Day No.) | 1,2,3,5,7 | 2,3,5,7 | 2,3,5 |
| Increased leukocytes (Day No.) | 2 | - | - |
| Decreased leukocytes (Day No.) | 5 | - | - |
| Increased IL-6 (Day No.) | - | - | - |
| Decreased IL-6 (Day No.) | 1,2,3,5,7 | 1,2,3 | 1,2,3 |
